# The “oral” history of COVID‐19: Primary infection, salivary transmission, and post‐acute implications

**DOI:** 10.1002/JPER.21-0277

**Published:** 2021-09-07

**Authors:** Julie Teresa Marchesan, Blake M. Warner, Kevin Matthew Byrd

**Affiliations:** ^1^ Division of Comprehensive Oral Health, Adams School of Dentistry University of North Carolina at Chapel Hill North Carolina; ^2^ Salivary Disorders Unit, National Institute of Dental and Craniofacial Research National Institutes of Health Bethesda Maryland; ^3^ Department of Innovation & Technology Research ADA Science & Research Institute Gaithersburg Maryland

**Keywords:** COVID‐19, oral cavity, oral pathology, oropharynx, saliva, SARS‐CoV‐2, virology

## Abstract

Severe acute respiratorysyndrome coronavirus 2 (SARS‐CoV‐2), the causative agent of COVID‐19, has led to more than 3.25 million recorded deaths worldwide as of May 2021. COVID‐19 is known to be clinically heterogeneous, and whether the reported oral signs and symptoms in COVID‐19 are related to the direct infection of oral tissues has remained unknown. Here, we review and summarize the evidence for the primary infection of the glands, oral mucosae, and saliva by SARS‐CoV‐2. Not only were the entry factors for SARS‐CoV‐2 found in all oral tissues, but these were also sites of SARS‐CoV‐2 infection and replication. Furthermore, saliva from asymptomatic individuals contained free virus and SARS‐CoV‐2‐infected oral epithelial cells, both of which were found to transmit the virus. Collectively, these studies support an active role of the oral cavity in the spread and transmission of SARS‐CoV‐2 infection. In addition to maintaining the appropriate use of personal protective equipment and regimens to limit microbial spread via aerosol or droplet generation, the dental community will also be involved in co‐managing COVID‐19 “long haulers”—now termed Post‐Acute COVID‐19 Syndrome. Consequently, we propose that, as SARS‐CoV‐2 continues to spread and as new clinical challenges related to COVID‐19 are documented, oral symptoms should be included in diagnostic and prognostic classifications as well as plans for multidisciplinary care.

## 1 INTRODUCTION

The emergence of SARS‐CoV‐2 as a novel human pathogenin December 2019 has changed our lives irrevocably. For many of us in the research and the clinic, this virus has exposed unknown vulnerabilities, highlighted overlooked areas of investigation, and challenged long‐held assumptions about the oral cavity. Although this has been a period of great challenge for all, the catalyst of COVID‐19 has illuminated concepts critical to patient care and hopefully opened a permanent seat at the table for oral health care in global disease discussions.

For example, since the beginning of the COVID‐19 pandemic, the oral cavity has been minimally considered in COVID‐19 pathogenesis, often presented as a passive conduit for the transmission of SARS‐CoV‐2 from other parts of the respiratory tract. However, COVID‐19 patients present with highly variable signs and symptoms, modes of transmission, and severity—each of which affects disease diagnosis and treatment. This includes a wide array of oral signs and symptoms.

Our teams, primarily from the National Institutes of Health, the Wellcome Sanger Institute, and the University of North Carolina at Chapel Hill teamed up to broadly ask the question of whether SARS‐CoV‐2 infection could occur within the oral cavity. Surprisingly, we found that all major oral mucosal sites and salivary glands were at risk for infection. Furthermore, we wondered about the potential for SARS‐CoV‐2 transmission via saliva and saliva as a diagnostic. We found that asymptomatic individuals can transmit SARS‐CoV‐2 via saliva, and salivary SARS‐CoV‐2 levels correlate with taste alterations. We also showed that both saliva and blood can be used to test for the presence of antibodies against SARS‐CoV‐2.

In this commentary, we acknowledge past research that supported our hypothesis, vast efforts that led to these findings in the pandemic and summarize the implications of these findings. This is the “oral history” of the oral health research community's most impactful efforts. We also want to focus this community on the variable post‐acute COVID‐19 syndrome (“long COVID” or “long haulers”). This is an emerging condition (1) in which initial disease severity is not necessarily predictive of post‐acute COVID‐19 symptoms, (2) that affects females more often than males, (3) can include oral symptoms. Simply put, this is a serious public health crisis and should not be ignored.

## 2 HYPOTHESIS: AN ORAL‐SYSTEMIC AXIS IN COVID‐19

As of May 2021, coronavirus disease 2019 (COVID‐19) remains a global public health emergency. This designation is the first since the declaration of the H1N1 influenza pandemic in 2009. Severe acute respiratory syndrome coronavirus 2 (SARS‐CoV‐2), the causative agent of COVID‐19, has led to more than 3.25 million deaths worldwide as of May 2021. COVID‐19 is now characterized by its variability—whether by signs, symptoms, spread, severity, or sequelae.^1^ Another challenge has emerged with “variants of concern” appearing across the world, with greater transmission and/or immune evasion potential.

**FIGURE 1:**
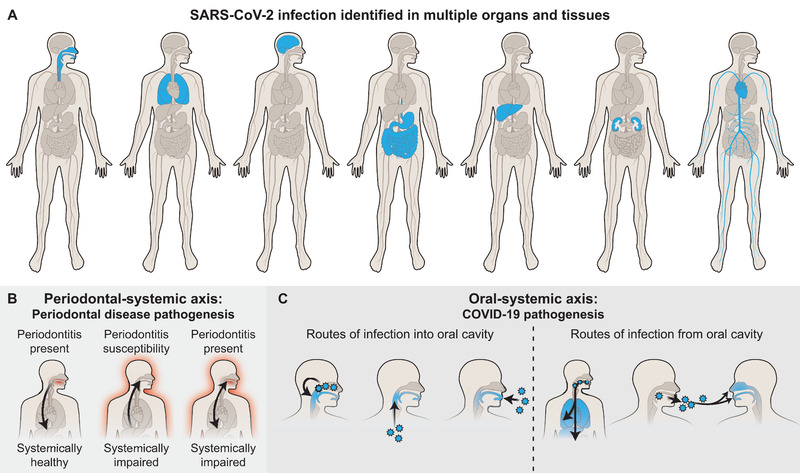
**The oral‐systemic axis of SARS‐CoV‐2**. (**A**) Infection can occur in multiple tissues, including the nasal cavity, oral cavity, intestines, brain, cardiovascular system, liver, and kidneys. (**B**) Periodontal medicine represents a hypothetical, bidirectional relationship between oral and systemic health through low‐grade delivery of microorganisms, hyperactive immune cells, and proinflammatory cytokines. The red glow depicts an inflammatory condition with arrows indicating the direction of the interaction between the oral cavity and the rest of the body. (**C**) These concepts of oral‐systemic connection apply to COVID‐19/SARS‐CoV‐2. Viral particles move from sites of existing infection to new sites of infections as indicated by the blue color. The oral cavity can become infected by viral entry through the nasal‐oral axis, the pulmonary‐oral axis, or the external environment. Infection established in the oral cavity can transmit SARS‐CoV‐2 within the same individual to the gastrointestinal tract or the lungs and can transmit the virus to another individual via saliva. [Credit: Heather McDonald, BioSerendipity, LLC, Elkridge, MD]

The oral cavity has been minimally considered in COVID‐19 pathogenesis, often presented as a passive conduit for the transmission of SARS‐CoV‐2 from other parts of the respiratory tract. By April 2020, the published symptom lists suggested the potential for direct infection of the brain, eyes, nose, lungs, cardiovascular system, liver, kidneys, and intestines (Figure 1A). At that time, there was no published evidence for direct infection of the oral cavity by SARS‐CoV‐2, despite evidence for free virus in saliva that led to an FDA emergency use authorization for it as a COVID‐19 diagnostic.^2^

However, humans constantly encounter airborne particles throughout the entire respiratory system, including the inter‐connected lungs, nasal, and oral cavity. Therefore, we hypothesized that all three sites—lungs, nasal cavity, and oral cavity—were infected by SARS‐CoV‐2 and that oral secretions had a role in viral transmissibility. These ideas were bolstered by over a century of evidence that supported the oral‐systemic axis concept in which the oral health or disease and the systemic health or disease are inter‐connected; substantiated research since the late 1980s has placed periodontal disease in the center of this link.^3^, ^4^ The bidirectional relationship, originally defined by Steven Offenbacher as “periodontal medicine”, suggested that the oral cavity could serve as a source of transient bacteremia and a low‐grade systemic inflammation (Figure 1B).^5^ At the time, this was a novel concept that proposed an integrated medical‐oral care team and aimed to remove the artificial barrier between professional health domains.

Numerous lines of evidence now support a role for the oral cavity in microbial transmission throughout the human body (Figure 1B, C). Many viruses can infect the oral mucosa and salivary glands, including herpesviruses, retroviruses, cytomegaloviruses, influenza viruses, and others.^6^ Non‐human primate studies on the first SARS pandemic suggested that the oral cavity was at risk for infection by SARS‐CoV‐2.^7^ Furthermore, viral transmission from the oral cavity into other organs and other individuals is supported by the many viruses found in saliva, as well as the physiology of the human ‘inhalation interface’, which includes multiple tissues and their niche‐secreted fluids with microbes, cytokines, and cells.^8^

Considering the potential for oral‐systemic interactions in bacterial and viral diseases like COVID‐19, it has recently been shown that the oral microbiome can be distributed more commonly along the respiratory and gastrointestinal tracts than previously assumed.^9^. Furthermore the idea of an oral‐systemic “transmission axis” has been recently explored in gut^10^ and lung diseases^11^; however, whether the oral microbiome can influence the distant sites and exacerbate COVID‐19 progression remains speculative. Although the oral‐systemic axis is hypothetical, this scientific history combined with numerous pandemic reports supported the rationale for exploring whether the oral cavity played a role in COVID‐19 pathogenesis (Figure 1C).

## 3 COVID‐19 OF THE ORAL CAVITY: DIRECT INFECTION OR SECONDARY MANIFESTATION?

### 3.1 Discovery of broad oral infection by SARS‐CoV‐2

**FIGURE 2:**
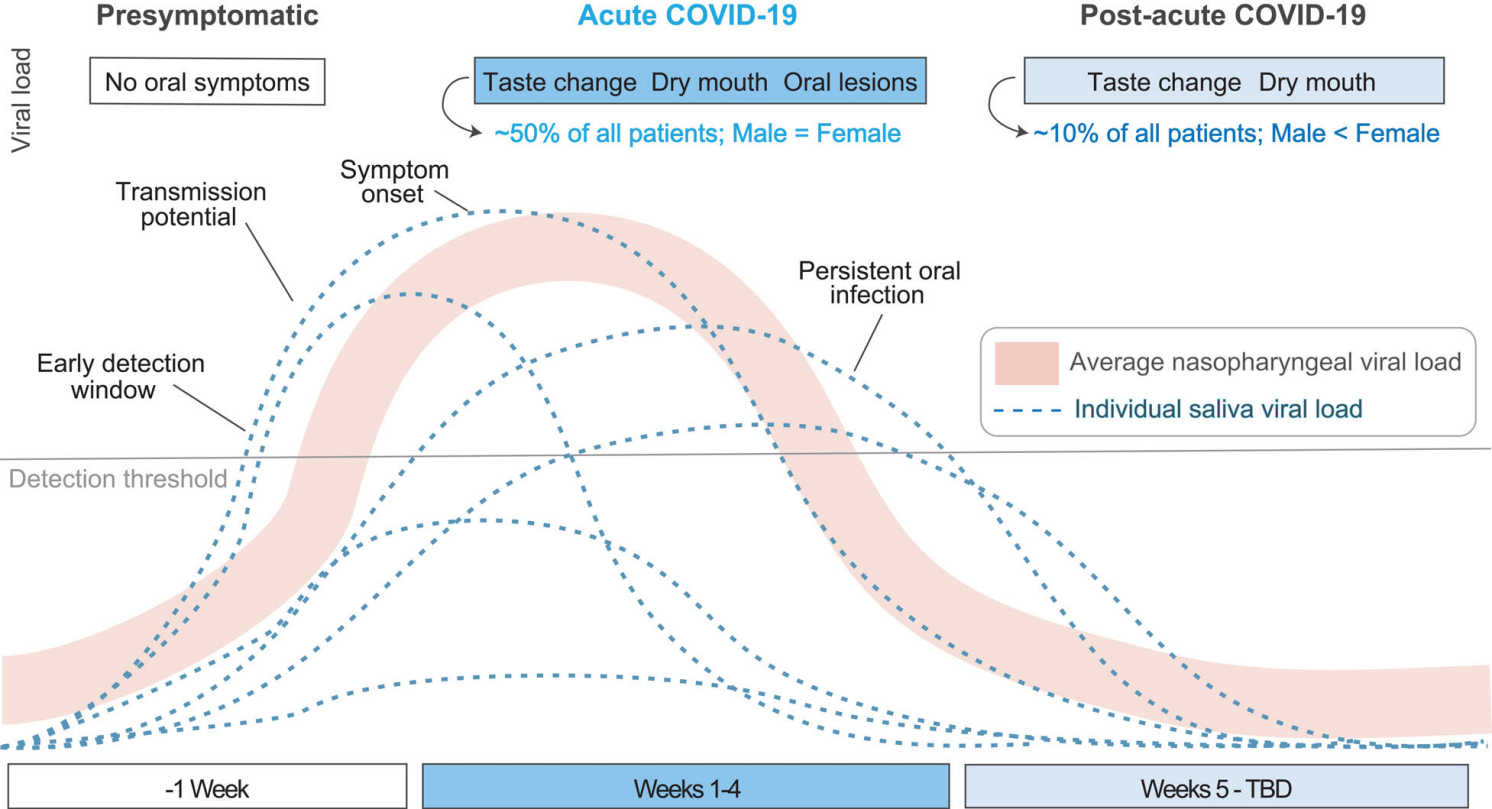
**Viral kinetics of SARS‐CoV‐2 in saliva**. Graph of viral kinetics in saliva of individuals and the average viral kinetics detected by nasopharyngeal testing. In some individuals, oral infection provides an early detection and transmission window that may precede symptom onset. Oral symptoms associated with acute and post‐acute COVID‐19, along with their relative prevalence in males and females, are shown at the top. Post‐acute COVID‐19 syndrome (‘long COVID’ or ‘long haulers’) affects females more often than males, estimated at a 4:1 ratio and can include oral symptoms

Despite the potential for direct oral tissue infection by SARS‐CoV‐2 and the oral symptoms in acute COVID‐19 (Table 1), the challenge has been to distinguish causation from correlation. Critical questions related remained as to whether the various oral symptoms were because of infection oral tissues by SARS‐CoV‐2 or if these COVID‐19 manifestations were related to generalized, severe inflammation from an infection elsewhere in the body. Additionally, many groups were interested in the potential infectiousness of saliva (Figure 1C). The resolution of these questions required extensive collaboration between oral health care scientists and providers, medical doctors, basic scientists, and bioinformaticians. The implications for comparing oral and nasopharyngeal viral load concordance^12^ as well as discovering primary oral infection by SARS‐CoV‐2 are relevant for testing in the pre‐symptomatic stage, identifying symptomatic infection, and providing post‐acute COVID‐19 patient care (Figure 2).

**TABLE 1:** Reported oral COVID‐19 symptoms. The symptoms described are based on multiple reports; strength of evidence for direct oral tissue infection highlighted by weak (*) to strong (****). These rankings do not account for secondary manifestations of the disease or side effects of pharmacotherapies for COVID‐19

Oral symptoms	Sites
Taste loss or alterations (ageusia, hypogeusia, dysgeusia)	Tongue ^****^
Papillitis, glossitis (also known as “COVID tongue”)	Tongue ^***^
White‐coated tongue	Tongue ^*^
Mucositis, pinpoint ulcerations, erythema	Oral mucosa ^**^
Mucosal plaques	Oral mucosa ^*^
Mucosal desquamation	Oral mucosa ^**^
Enanthema, petechiae	Oral mucosa ^*^
Pustules, blisters, vesicles, macula, hyperpigmentation	Oral mucosa ^*^
Aphthous stomatitis	Oral mucosa ^*^
Gingivitis	Periodontium ^*^
Necrotizing periodontal disease	Periodontium ^*^
Parotitis or sialadenitis	Salivary glands ^****^
Xerostomia or hyposalivation	Salivary glands ^****^
Trigeminal neuralgia	Neurologic ^*^
Facial tingling	Neurologic ^*^
Temporomandibular joint abnormalities	Neurologic ^*^
Candidiasis (also known as thrush)	Generalized ^*^
Burning mouth or burning, itching sensations (pruritus)	Generalized ^*^

**FIGURE 3:**
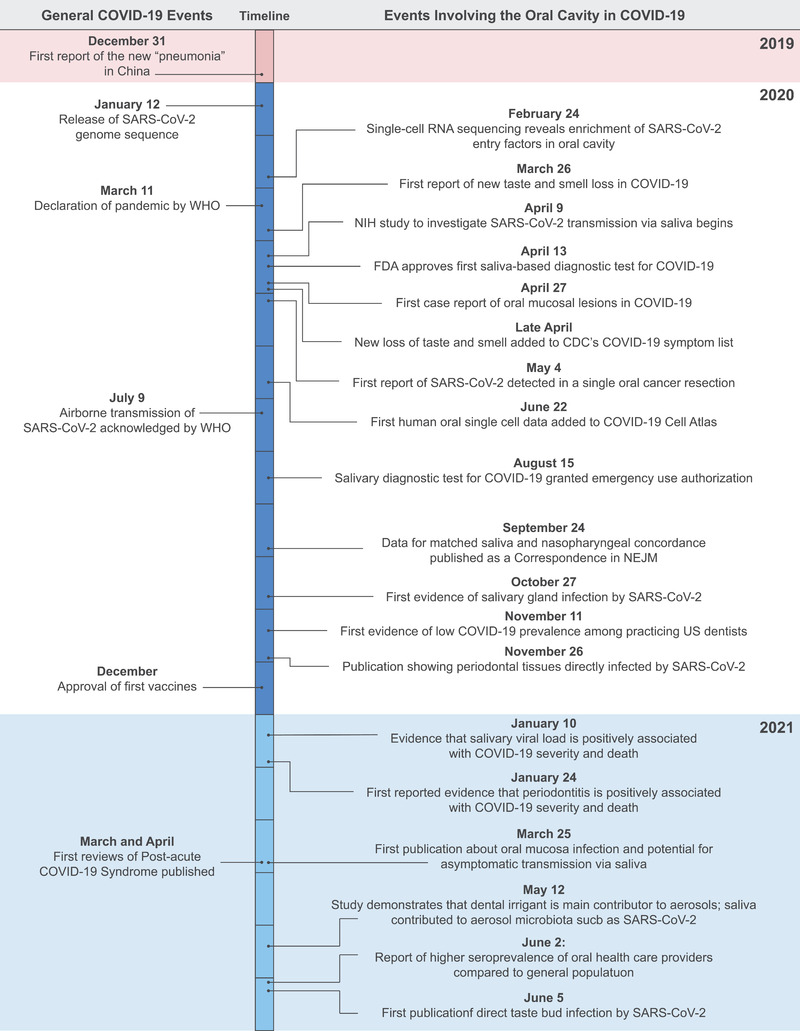
**An “oral” history of COVID‐19**. The left side shows selected general events related to the SARS‐CoV‐2 pandemic. The right side shows selected key events related to the oral cavity as a key site for infection, prevention, diagnosis, transmission, risk, and immunity. Neither side is intended to be comprehensive. [Credit: Heather McDonald, BioSerendipity, LLC, Elkridge, MD]

The highlighted findings and new data presented here build off incredible efforts from the international research community and funding organizations who supported the rapid study of SARS‐CoV‐2 since December 2019 (Figure 2). The dissemination of COVID‐19 discoveries by scientists to other scientists and the public has been dynamic, and synthesizing relevant evidence requires listening to patient experiences, reading news sources, and collecting reports across hundreds of publications—including the exponential increase of preprints on bioRxiv and medRxiv. Given the urgent need to answer questions, the availability of preprint articles was critical to driving hypotheses and collaborations—with some appearing nearly a year before official publication in a peer‐reviewed journal.^13^ Multiple key findings were rapidly released and changed clinical recommendations (see: The “Oral” History of COVID‐19 Timeline in Figure 3).

An example of an oral finding that was shared rapidly and was critically important in driving healthcare decisions is represented by the rapid publication following anecdotal reports in March 2020 that health care providers recommended that patients stay home if they experienced a loss of taste or smell. The first report was published on this phenomenon as a preprint publication on March 26.^14^ This prompted the American Academy of Otolaryngology in late March to propose adding smell and taste alterations to COVID‐19 screening protocols. This update to the screening protocols turned out to be critical: The documented prevalence of taste alterations is now reported in nearly 50% of all COVID‐19 patients.^15^ Sudden changes to olfaction and gustation are more prevalent in this disease compared to other viral diseases and now serve as a key COVID‐19 symptom.^16^

Data indicating that the oral cavity could be a site of SARS‐CoV‐2 infection began to accumulate (Figure 3). The first case reports of oral mucosal lesions were published in late April 2020.^17^ A few weeks later, a study published using the COVID Symptom Study smartphone‐based app compiled data from 2,618,862 participants, and 65% of respondents reported taste loss.^18^ Further, this project demonstrated the future potential for crowdsourcing disease data in real‐time. One‐off reports were also published regarding high transmission rates from activities that involve the oral cavity such as singing in choir practice.^19^ At the same time, others published hypotheses that the oral cavity may be a site of direct infection in COVID‐19^20^ or that periodontal disease may serve as a risk factor for severe COVID‐19.^21^ By summer 2020, other less common oral symptoms were being reported more often in COVID‐19 patients, including salivary gland inflammation^22^ and oral mucosal lesions^23^ (Table 1). Despite the numerous lines of evidence in support of an oral‐COVID‐19 axis—including a case report that detected the virus in an oral cancer resection^24^—it remained unclear whether these oral manifestations were evidence of primary infection of the oral cavity by SARS‐CoV‐2 or secondary to COVID‐19‐related inflammation. We also note that these are not mutually exclusive and that some pharmacotherapies for COVID‐19 may also contribute to these reported oral signs and symptoms.

Data were needed to confirm that each reported oral manifestation results from SARS‐CoV‐2 infection, including the analysis of the viral entry factors in each oral tissue niche. Additionally, identification of infection in situ is critical. By early February 2020, one receptor for SARS‐CoV‐2 cellular entry was known: angiotensin‐converting enzyme 2 (ACE2).^25^ This rapid discovery was possible because previous researchers had determined that SARS‐CoV, the causative agent of the 2003 SARS pandemic, used ACE2 to enter cells.^26^ Weeks later, Xu et al. published a single‐cell transcriptomic analysis of *ACE2* expression in the human tongue, gingiva, and buccal mucosal cells that suggested the tongue was especially at risk for direct infection by SARS‐CoV‐2.^27^ Although these studies provided evidence for oral mucosal risk for infection the salivary glands were not included in this study. Subsequent reports suggested that the salivary glands were similarly at risk and, if infected, may serve as a primary cause of asymptomatic transmission.^28^ Based on these early studies and the heterogeneity of oral epithelial cells in distinct niches,^29^ we hypothesized some oral cell types in the mucosa and the glands would be especially susceptible to infection.

**FIGURE 4:**
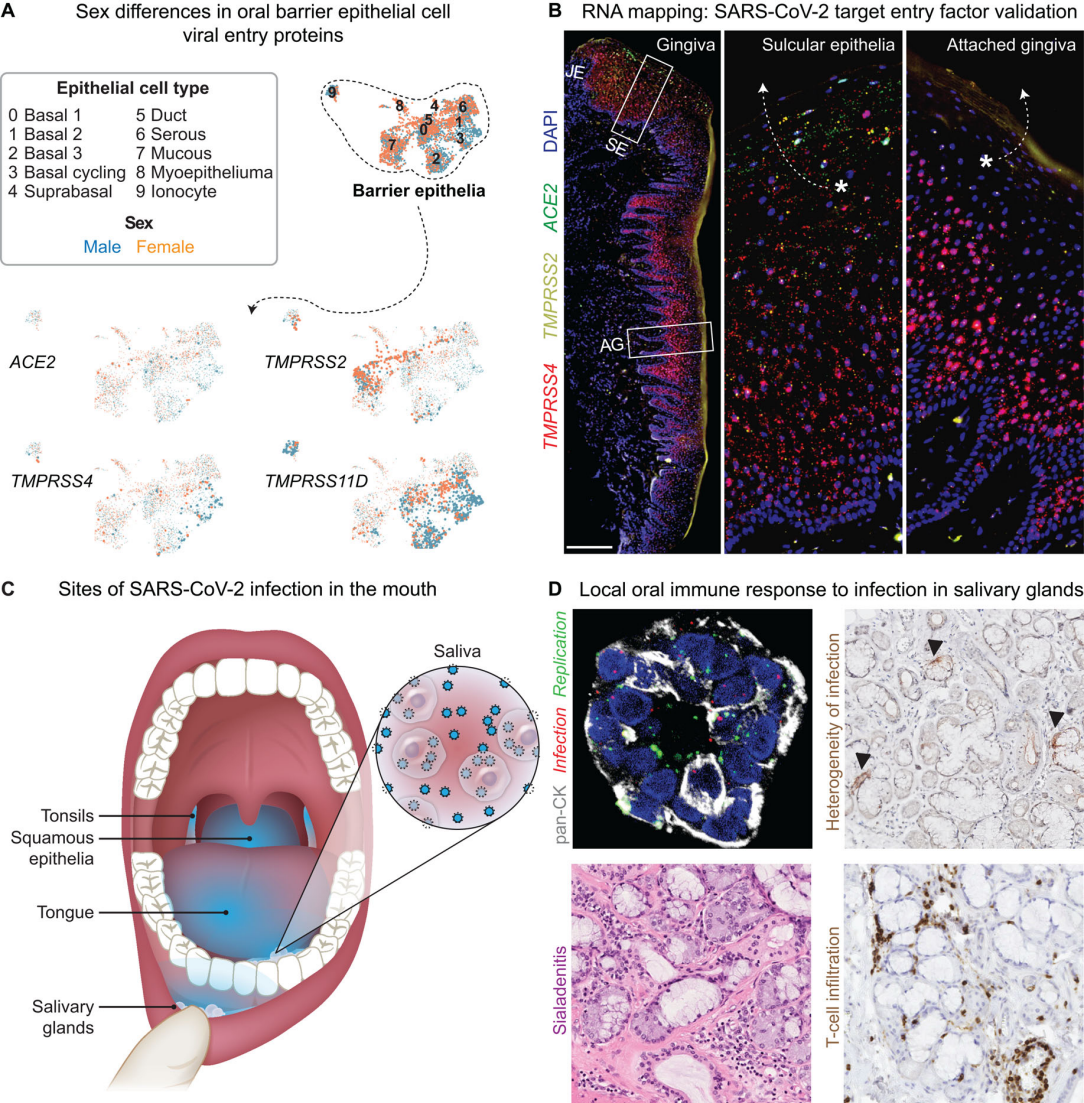
**Evidence for broad oral tissue infection by SARS‐CoV‐2**. (**A**) To guide the single‐cell mapping of SARS‐CoV‐2 infection by cell type, we generated and integrated single‐cell RNA sequencing datasets from gingiva, mucosa, and minor salivary glands (9 samples; 13,824 cells). Of the 34 unique cell types (epithelial, mesenchymal, and immune cell populations), the only cells that expressed the indicated genes involved in SARS‐CoV‐2 entry were barrier epithelial cells. The abundance of the indicated transcripts within each of the barrier epithelia populations colored according to their presence in males and females is shown. See Huang et al. for detailed methods.^32^ (**B**) Visualization of mRNA for the indicated SARS‐CoV‐2 entry factors was performed with RNAscope ® (ACD) in situ hybridization of healthy human gingival tissues. The transcripts for the indicated entry factors were enriched in junctional epithelia (JE) and sulcular epithelia (SE), especially in the most terminally differentiated and shedding populations (indicated with asterisks) in the gingiva and oral mucosa. AG, attached gingiva. (See Methods for details.) (**C**) The sites of SARS‐CoV‐2 infection in the oral cavity. Infection is indicated by the blue glow. Only sites of known infection are labeled. (**D**) SARS‐CoV‐2 infection and the immune response in a human salivary gland. Epithelial cells (ducts and acini) were detected with an antibody to all cytokeratins (pan‐CK); infection and replications with in situ hybridization (red and green, respectively). The heterogeneity of gland infection was shown with immunohistochemistry, and arrowheads indicate spike protein staining. Sialadenitis was diagnosed by a team of pathologists; infiltrating T cells were detected with a clinical antibody panel. [Credit for panel C: Heather McDonald, BioSerendipity, LLC, Elkridge, MD]

Most groups studying SARS‐CoV‐2 host entry factors focused on ACE2, the TMPRSS protease family (TMPRSS2, TMPRRS4, and TMPRSS11D), and cathepsins (CTSB and CSTL).^30^ The power of collaboration was evident in the multiple published studies by groups associated with the Human Cell Atlas (HCA). These international collaborations integrated single‐cell transcriptomic assays from multiple tissues to identify cell‐specific vulnerabilities for infection by SARS‐CoV‐2 throughout the body,^31^ resulting in the COVID‐19 Cell Atlas (https://www.covid19cellatlas.org/). As part of this effort, our team donated single cell RNA sequencing datasets of human gingiva and salivary glands to this atlas in June 2020, leading to the creation of the Oral & Craniofacial Biological Network (HCA‐OCBN) in September 2020. Using our group's single cell atlases, we found broad expression of *ACE2* and genes encoding TMPRSS family members in barrier epithelial subpopulations.^32^ Here, we show the results of the examination of the sex‐specific expression of these viral entry host genes (Figure 4A). Our results indicate that the expression of these entry factor‐encoding genes was similar in epithelial cells from males and females, despite the higher burden of acute COVID‐19 in older males with defined comorbidities.^33^ Whether the oral host response to the viral infection is distinct between sex is still unexplored.

For low abundance transcripts like those for ACE2, single‐cell transcriptomics analyses often serve as a relative guide rather than an absolute measurement. We estimate that low abundance transcripts could be underestimated by a range of 1 in 5 to 10 transcripts per cell (unpublished observations). To evaluate specific entry factors with greater sensitivity, we designed probes to map these entry factor transcripts throughout the oral epithelia sites using multiple techniques including in situ hybridization.^32^ Based on these analyses,^32^ we conclude that (1) all oral mucosae and glands are at risk for infection by SARS‐CoV‐2, and (2) the most at‐risk epithelial cells are those exposed to the external environment. These highly at‐risk epithelial cells include the gingiva, where *ACE2*, *TMPRSS2*, and *TMPRSS4* were enriched in the sulcular and junctional epithelia (Figure 4B) and are the same cells that constantly shed into saliva and may stick to medical gloves when performing an oral exam (Figure 4C). To determine if these sites were infected, we obtained specimens from the oral cavity from the NIH COVID‐19 Autopsy Consortium. Using multiple assays, we found infection and viral replication of the major and minor salivary glands and oral mucosae (Figure 4C, D).^32^

Finding viral entry factors, infection, and viral replication within multiple tissues of the oral cavity provides strong evidence that some of the reported oral COVID‐19 manifestations are likely because of direct infection by SARS‐CoV‐2. Many of the oral COVID‐19 manifestations relate to inflammation (Table 1). Indeed, tissue damage and cell death, mediated by T cells and B cells, occurs in response to SARS‐CoV‐2 infection.^34^ Here, we show data of the local oral immune response to salivary gland infections by SARS‐CoV‐2 (Figure 4D). The infected glands exhibited pathologic signs of sialadenitis (salivary gland inflammation) that was associated with T cell infiltration (see Huang et al.^32^). Though we found oral mucosal infection of a non‐keratinized site, the salivary glands are the only confirmed oral niche to date, that has been proven to be the result of primary infection. To do this required discovery that 1) the glands and mucosa express genes encoding host factors that mediate viral entry and 2) that these sites were confirmed to be a site of SARS‐CoV‐2 infection and replication. However, only the glands were the only sites identified and characterized by a robust host immune response at the site of infection.

These findings underscore the importance of further research to understand the consequences of SARS‐CoV‐2 infection in other oral niches. For example, the first report of direct infection of the taste papillae was just published, suggesting a substantial effect on the epithelial progenitor population.^35^ Determining correlation or causation for other oral manifestations still requires an organized and motivated research community to share research and resources. Open questions include how these oral niche susceptibilities change during childhood development into adulthood, how chronic oral inflammatory conditions influence viral infection and systemic disease progression, which of the reported oral conditions are a direct result of viral infection of the oral tissues, and in those infected sites, what impact SARS‐CoV‐2 infection had on those tissues long‐term.

### 3.2 Salivary transmission, antibody response, and predictive potential

In April 2020, one of the first COVID‐19 clinical trials (NCT04348240) at the NIH was started. This trial aimed to evaluate the transmissibility and viral load of SARS‐CoV‐2 from saliva and the nasopharynx.^32^ This study was also part of our oral‐COVID‐19 work, recruiting acute COVID‐19 and at‐risk cohabitating subjects and monitoring them both prospectively. We found that saliva from individuals with mild COVID‐19 or even asymptomatic individuals contained substantial concentrations of free virus mixed with infected cells shed from the oral mucosae, suggesting that the oral cavity alone may be a source of viral transmissibility within and between individuals. This saliva also was monitored prospectively for IgG antibodies, with the results closely matching blood antibody profiles and levels, suggesting the potential for neutralization against SARS‐CoV‐2 in most subjects by 3 weeks post‐diagnosis. These results further demonstrate the potential for non‐invasive oral sampling as a method for studying systemic immunity in COVID‐19 and for testing for SARS‐CoV‐2 infection.^32^

Additionally, a cohort of NIH employee saliva samples was studied, revealing that some individuals who remained asymptomatic could display high saliva viral loads (measured by CT values as high as < 20). These values could be higher than symptomatic patients. The saliva from these asymptomatic individuals was discovered to contain both free virus and the SARS‐CoV‐2‐infected epithelial cells; both were capable of transmitting SARS‐CoV‐2 in culture. Overall, these preliminary data show that the oral cavity represents a robust and underappreciated site for SARS‐CoV‐2 infection and raises the possibility that the oral cavity actively participates in viral transmission, even in asymptomatic individuals.

## 4 CLINICAL IMPLICATIONS OF SARS‐COV‐2 FINDINGS IN THE ORAL MUCOSA AND SALIVA

Although the nasal axis for SARS‐CoV‐2 infection dominated early research reports, our published and preliminary findings reported here provide multiples lines of evidence for a parallel oral axis in both SARS‐CoV‐2 infection and transmission. This evidence includes the discovery of direct oral infection by SARS‐CoV‐2 in every major oral tissue niche, detection of a robust immune response at sites of infection, and determination that saliva contains infectious virus and thus may play an important role in SARS‐CoV‐2 transmission. Whereas our clinical studies of COVID‐19 subjects included ambulatory asymptomatic or mildly symptomatic COVID‐19 subjects, other recent studies performed complementary analyses of severe COVID‐19 subjects. A recent preprint from Silva et al. reports a “unifying” set of parameters in severe COVID‐19, which includes a correlation between salivary viral load and COVID‐19 disease severity.^36^ A lower salivary viral load was associated with better clinical outcomes; contrastingly, higher viral load was associated with pro‐inflammatory effector cytokine expression, immune dysregulation, and death. These outcomes were not correlated with nasopharyngeal viral load, emphasizing the centrality of the oral axis in COVID‐19 and further establishing rationale for research into the various upper airway sampling methods (nasal/nasopharyngeal, saliva, oropharyngeal swabs, bronchoalveolar lavage, etc.) for other infectious and chronic inflammatory diseases.

Our data support the potential for saliva transmission to contribute to the asymptomatic and pre‐symptomatic transmission of SARS‐CoV‐2—the so‐called “Achilles's heel” of this pandemic.^37^ This is further emphasized by a University of Colorado study of more than 70,000 saliva samples in the asymptomatic or pre‐symptomatic stage, finding 2% were positive for SARS‐CoV‐2—but at similar viral loads as symptomatic individuals in the range of potential transmission.^38^ We hypothesize that most community spread occurred in these asymptomatic or pre‐symptomatic stages via saliva/respiratory‐driven airborne transmission. This phenomenon is likely important for a variety of other viral diseases, though much work remains to be done. Other questions have remained about the risk for oral health care workers in the clinic if saliva is potentially infectious fluid. Work from Meethil et al. addressed this in an original study of dental aerosols from hygiene restorative, and implant procedures,^39^ concluding that the majority of procedure‐derived aerosol microbiota were from the irrigant—not from saliva. This includes aerosols from SARS‐CoV‐2 positive patients.

These data support the continued use of common sense guidelines, such as the appropriate use of personal protective equipment and regimens to decrease the microbial load in aerosols and to reduce droplet generation during dental procedures (reviewed in detail here^40^). Real‐world data for the oral health care community show that dentists and hygienists, fortunately, have low rates of SARS‐CoV‐2 infection.^41^ These findings emphasize the safety of professional oral health care visits and indicate that low rates of infection are tied to the widespread adoption of risk‐mitigating protocols in the dental operatory. However, recent data from a report from oral health care providers in the UK found an increased rate of SARS‐CoV‐2 antibodies when compared to the surrounding community, suggesting that some providers and staff in dental clinics are at increased susceptibility to infection.^42^ More research is required across the globe to understand the impact of risk‐mitigating measures and the hazards of the dental clinic for pathogen transmissibility among oral health care workers.

### 4.1 Post‐acute COVID‐19

Highlighting the uniqueness of COVID‐19 in relation to other viral diseases, it is reported that some individuals will develop post‐acute COVID‐19 syndrome (formerly “long‐COVID” or “long haulers”). Although we acknowledge that this is a developing situation, recent efforts are starting to focus on this condition,^43^ and like COVID‐19, its sign and symptoms are surprisingly varied., Clinicians must recognize the diversity of persistent signs and symptoms to better approach our patients with knowledge and empathy. Symptoms of post‐acute COVID‐19 syndrome include fatigue, headache, shortness of breath, cognitive effects, chest pain, kidney disease, and new hair loss.^44^ Furthermore, ∼10% of patients have persistent taste or smell alterations at 6‐month post‐diagnosis, this persistent alteration in taste or smell is particularly relevant because about 1/3 of all COVID‐19 patients report neurological or psychiatric diagnoses 6 months after diagnosis.^45^

Post‐acute COVID‐19 syndrome is reported to affect ∼10% of patients 1‐month post‐diagnosis, about 5% of patients at 2‐months, 2.5% greater than 3 months.^44^ Furthermore, a recent report summarizing a 100 patient cohort at the Mayo Clinic found most long haulers were not formerly hospitalized in the acute phase and did not present with pre‐existing conditions prior to infection by SARS‐CoV‐2.^46^ Initial disease severity is not necessarily predictive of post‐acute COVID‐19 syndrome. Simply put, this is a serious public health crisis and should not be ignored. With nearly 200,000,000 COVID‐19 cases worldwide, 5 million subjects will currently be affected by this disease greater than 6 months after the initial acute phase. Whether they will recover is still to be seen. Those most likely to develop post‐acute COVID‐19 were older individuals, those with higher body mass index, and/or those who experienced five or more acute COVID‐19 symptoms.^44^ Surprisingly, even among patients who experienced originally an “asymptomatic” or mildly symptomatic disease course, ∼20% report persistent or newly emergent symptoms 1‐month post‐diagnosis.^47^ Although males test positive for COVID‐19 more often and display more severe disease, female sex is a predictor of post‐acute COVID‐19 syndrome with one study showing a 4:1 burden of the disease for females across 56 countries.^48^

Although we do not propose that taste and smell alterations are sole drivers of such neurological or psychiatric outcomes, taste and smell alterations are associated with poorer mental health.^49^ Consequently, such sensory disturbances may create a positive feedback loop whereby this newly defined “disabled” patient requires additional care through multidisciplinary co‐management. For example, long‐term olfactory and gustatory alterations have been associated with damage in the brain.^50^ Furthermore, the chronic effects of post‐acute COVID‐19 syndrome, such as tissue damage or depression, establish it as a potential risk modifier for periodontal disease. Clinicians must be aware and stay informed of these interconnected conditions as there is much more for us to learn.

### 4.2 Other emerging clinical implications

Despite the intensive and rapid research efforts during this pandemic, many unknowns remain. We have had little time to understand the full clinical manifestations of COVID‐19. The first patients were identified in December 2019 and the first manuscripts about staging patients with COVID‐19 were not published until the end of 2020. To better serve clinicians and patients, more work is needed to understand the importance of antimicrobial mouth rinses, the possibility of periodontal disease exacerbation after COVID‐19, and the prevalence and severity of orofacial adverse effects after vaccination. Finally, with rapid diagnostic tests for SARS‐CoV‐2 infection continuing to be approved and the possibility of detecting neutralizing antibodies in saliva after vaccination, there is a glimmer of hope regarding a future collaborative paradigm between oral and general medicine in which connected electronic patient records enable dental chairside diagnostics to be leveraged for improved precision health care.

## 5 METHODS

Detailed methodology for single‐cell transcriptomic analysis and immunohistochemistry/in‐situ hybridization (IHC/ISH) analyses (Figure 4A and 4D, respectively) is described in Huang et al.^32^ For the new data presented in Figure 4B, RNAscope® ISH (ACD) was performed following manufacturer's instruction; however, pretreatment conditions were modified: (1) protease plus digestion at 40 °C was implanted for 15 min and (2) antigen retrieval in RNAscope target retrieval in slide steamer control temperature at 99 °C for 15 min. Probes from ACD: ACE2 (848561); TMPRSS2 (848151) TMPRSS4 (470341; positive control (320861, 310041); negative control (320871, 310043).

## CONFLICTS OF INTEREST

Although the authors view each of these as non‐competing financial interests, we report that, in the last year, KMB has been a Scientific Advisor at Arcato Laboratories; KMB, BMW, and JTM are active members of the Human Cell Atlas.

## AUTHOR CONTRIBUTIONS

Conceptualization: Julie Teresa Marchesan, Kevin Matthew Byrd; Data Acquisition, Analysis, or Interpretation: Julie Teresa Marchesan, Kevin Matthew Byrd, Blake M. Warner; Writing – Original Draft: Julie Teresa Marchesan, Kevin Matthew Byrd; Critical Revision of the Manuscript: Julie Teresa Marchesan, Kevin Matthew Byrd, Blake M. Warner.

## Supporting information

SUPPLEMENTAL REFERENCES
