# Supplementary material for: The “oral” history of COVID‐19: Primary infection, salivary transmission, and post‐acute implications
Source: J Periodontol. 2021 Sep 7;92(10):1357–67. doi: 10.1002/JPER.21-0277 (PMC9374061; doi:10.1002/JPER.21-0277)
Supplement: Supplementary file 1 — SUPPLEMENTAL REFERENCES [file JPER-92-1357-s001.docx]

**SUPPLEMENTAL REFERENCES**

51. Chern A, Famuyide AO, Moonis G, Lalwani AK. Sialadenitis: A Possible Early Manifestation of COVID-19. *Laryngoscope* 2020;130:2595-2597.

52. Jimenez-Cauhe J, Ortega-Quijano D, de Perosanz-Lobo D, et al. Enanthem in Patients With COVID-19 and Skin Rash. *JAMA Dermatology* 2020.

53. dos Santos JA, Normando AGC, da Silva RLC, et al. Oral mucosal lesions in a COVID-19 patient: new signs or secondary manifestations? *International Journal of Infectious Diseases* 2020.

54. Guerini-Rocco E, Taormina SV, Vacirca D, et al. SARS-CoV-2 detection in formalin-fixed paraffin-embedded tissue specimens from surgical resection of tongue squamous cell carcinoma. *J Clin Pathol* 2020.

55. Zhou P, Yang XL, Wang XG, et al. A pneumonia outbreak associated with a new coronavirus of probable bat origin. *Nature* 2020;579:270-273.

56. Kuhn JH, Li W, Choe H, Farzan M. Angiotensin-converting enzyme 2: a functional receptor for SARS coronavirus. *Cell Mol Life Sci* 2004;61:2738-2743.

57. Xu H, Zhong L, Deng J, et al. High expression of ACE2 receptor of 2019-nCoV on the epithelial cells of oral mucosa. *Int J Oral Sci* 2020;12:8.

58. Xu J, Li Y, Gan F, Du Y, Yao Y. Salivary Glands: Potential Reservoirs for COVID-19 Asymptomatic Infection. *Journal of dental research* 2020;99:989.

59. Pascolo L, Zupin L, Melato M, Tricarico PM, Crovella S. TMPRSS2 and ACE2 Coexpression in SARS-CoV-2 Salivary Glands Infection. *Journal of dental research* 2020;99:1120-1121.

60. Caetano AJ, Yianni V, Volponi A, Booth V, D'Agostino EM, Sharpe P. Defining human mesenchymal and epithelial heterogeneity in response to oral inflammatory disease. *eLife* 2021;10.

61. Krivanek J, Soldatov RA, Kastriti ME, et al. Dental cell type atlas reveals stem and differentiated cell types in mouse and human teeth. *Nature communications* 2020;11:1-18.

62. Byrd KM, Piehl NC, Patel JH, et al. Heterogeneity within Stratified Epithelial Stem Cell Populations Maintains the Oral Mucosa in Response to Physiological Stress. *Cell stem cell* 2019;25:814-829.e816.

63. Jones KB, Furukawa S, Marangoni P, et al. Quantitative Clonal Analysis and Single-Cell Transcriptomics Reveal Division Kinetics, Hierarchy, and Fate of Oral Epithelial Progenitor Cells. *Cell stem cell* 2019;24:183-192.e188.

64. Singh M, Bansal V, Feschotte C. A Single-Cell RNA Expression Map of Human Coronavirus Entry Factors. *Cell reports* 2020;32:108175.

65. Wei J, Alfajaro MM, DeWeirdt PC, et al. Genome-wide CRISPR Screens Reveal Host Factors Critical for SARS-CoV-2 Infection. *Cell* 2021;184:76-91.e13.

66. Sungnak W, Huang N, Becavin C, et al. SARS-CoV-2 entry factors are highly expressed in nasal epithelial cells together with innate immune genes. *Nature medicine* 2020.

67. Ziegler C, Allon SJ, Nyquist SK, et al. SARS-CoV-2 receptor ACE2 is an interferon-stimulated gene in human airway epithelial cells and is enriched in specific cell subsets across tissues. 2020.

68. Regev A, Teichmann SA, Lander ES, et al. The Human Cell Atlas. *eLife* 2017;6.

69. Teichmann S, Regev A. The network effect: studying COVID-19 pathology with the Human Cell Atlas. *Nat Rev Mol Cell Biol* 2020;21:415-416.

70. Li X, Xu S, Yu M, et al. Risk factors for severity and mortality in adult COVID-19 inpatients in Wuhan. *J Allergy Clin Immunol* 2020;146:110-118.

71. Dawes C. Estimates, from salivary analyses, of the turnover time of the oral mucosal epithelium in humans and the number of bacteria in an edentulous mouth. *Archives of oral biology* 2003;48:329-336.

72. Mathew D, Giles JR, Baxter AE, et al. Deep immune profiling of COVID-19 patients reveals distinct immunotypes with therapeutic implications. *Science (New York, NY)* 2020;369.

73. Doyle ME, Appleton A, Liu QR, Yao Q, Mazucanti CH, Egan JM. Human Type II Taste Cells Express ACE2 and are Infected by SARS-CoV-2. *The American journal of pathology* 2021.

74. Brann D, Tsukahara T, Weinreb C, Logan DW, Datta SR. Non-neural expression of SARS-CoV-2 entry genes in the olfactory epithelium suggests mechanisms underlying anosmia in COVID-19 patients. *BioRxiv* 2020.

75. Silva J, Lucas C, Sundaram M, et al. Saliva viral load is a dynamic unifying correlate of COVID-19 severity and mortality. *medRxiv* 2021:2021.2001.2004.21249236.

76. Shirazi S, Stanford CM, Cooper LF. Characteristics and Detection Rate of SARS-CoV-2 in Alternative Sites and Specimens Pertaining to Dental Practice: An Evidence Summary. *J Clin Med* 2021;10.

77. Gandhi M, Yokoe DS, Havlir DV. Asymptomatic transmission, the Achilles’ heel of current strategies to control COVID-19. In: Mass Medical Soc, 2020.

78. Yang Q, Saldi TK, Gonzales PK, et al. Just 2% of SARS-CoV-2-positive individuals carry 90% of the virus circulating in communities. *Proceedings of the National Academy of Sciences of the United States of America* 2021;118.

79. Meethil AP, Saraswat S, Chaudhary PP, Dabdoub SM, Kumar PS. Sources of SARS-CoV-2 and Other Microorganisms in Dental Aerosols. *Journal of dental research* 2021:220345211015948.

80. Kumar PS, Geisinger ML, Avila-Ortiz G. Methods to mitigate infection spread from aerosol-generating dental procedures. *J Periodontol* 2020.

81. Kambhampati AK, O'Halloran AC, Whitaker M, et al. COVID-19-Associated Hospitalizations Among Health Care Personnel - COVID-NET, 13 States, March 1-May 31, 2020. *MMWR Morb Mortal Wkly Rep* 2020;69:1576-1583.

82. Estrich CG, Gurenlian JR, Battrell A, et al. COVID-19 Prevalence and Related Practices among Dental Hygienists in the United States. *J Dent Hyg* 2021;95:6-16.

83. Estrich CG, Mikkelsen M, Morrissey R, et al. Estimating COVID-19 prevalence and infection control practices among US dentists. *Journal of the American Dental Association (1939)* 2020;151:815-824.

84. Shields AM, Faustini SE, Kristunas CA, et al. COVID-19: Seroprevalence and Vaccine Responses in UK Dental Care Professionals. *Journal of dental research* 2021:220345211020270.

85. Huang C, Huang L, Wang Y, et al. 6-month consequences of COVID-19 in patients discharged from hospital: a cohort study. *Lancet (London, England)* 2021;397:220-232.

86. Logue JK, Franko NM, McCulloch DJ, et al. Sequelae in Adults at 6 Months After COVID-19 Infection. *JAMA Netw Open* 2021;4:e210830.

87. Nalbandian A, Sehgal K, Gupta A, et al. Post-acute COVID-19 syndrome. *Nature medicine* 2021.

88. Wu CT, Lidsky PV, Xiao Y, et al. SARS-CoV-2 infects human pancreatic β cells and elicits β cell impairment. *Cell Metab* 2021.

89. Tang X, Uhl S, Zhang T, et al. SARS-CoV-2 infection induces beta cell transdifferentiation. *Cell Metab* 2021.

90. Taquet M, Geddes JR, Husain M, Luciano S, Harrison PJ. 6-month neurological and psychiatric outcomes in 236 379 survivors of COVID-19: a retrospective cohort study using electronic health records. *Lancet Psychiatry* 2021.

91. Health F. A Detailed Study of Patients with Long-Haul COVID. 2021.

92. Vanichkachorn G, Newcomb R, Cowl CT, et al. Post COVID-19 Syndrome (Long Haul Syndrome): Description of a Multidisciplinary Clinic at the Mayo Clinic and Characteristics of the Initial Patient Cohort. In: *Mayo Clinic Proceedings*: Elsevier, 2021.

93. Moreno-Pérez O, Merino E, Leon-Ramirez J-M, et al. Post-acute COVID-19 Syndrome. Incidence and risk factors: a Mediterranean cohort study. *Journal of Infection* 2021;82:378-383.

94. Cirulli ET, Schiabor Barrett KM, Riffle S, et al. Long-term COVID-19 symptoms in a large unselected population. *medRxiv* 2020:2020.2010.2007.20208702.

95. Peckham H, de Gruijter NM, Raine C, et al. Male sex identified by global COVID-19 meta-analysis as a risk factor for death and ITU admission. *Nature communications* 2020;11:6317.

96. Davis HE, Assaf GS, McCorkell L, et al. Characterizing Long COVID in an International Cohort: 7 Months of Symptoms and Their Impact. *medRxiv* 2020:2020.2012.2024.20248802.

97. Taalman H, Wallace C, Milev R. Olfactory Functioning and Depression: A Systematic Review. *Front Psychiatry* 2017;8:190.

98. Hur K, Choi JS, Zheng M, Shen J, Wrobel B. Association of alterations in smell and taste with depression in older adults. *Laryngoscope Investig Otolaryngol* 2018;3:94-99.

99. Gopinath B, Anstey KJ, Sue CM, Kifley A, Mitchell P. Olfactory impairment in older adults is associated with depressive symptoms and poorer quality of life scores. *Am J Geriatr Psychiatry* 2011;19:830-834.

100. Baharvand M, ShoalehSaadi N, Barakian R, Moghaddam EJ. Taste alteration and impact on quality of life after head and neck radiotherapy. *J Oral Pathol Med* 2013;42:106-112.

101. Ismail, II, Gad KA. Absent Blood Oxygen Level-Dependent Functional Magnetic Resonance Imaging Activation of the Orbitofrontal Cortex in a Patient With Persistent Cacosmia and Cacogeusia After COVID-19 Infection. *JAMA Neurol* 2021.

102. Rosania AE, Low KG, McCormick CM, Rosania DA. Stress, depression, cortisol, and periodontal disease. *Journal of periodontology* 2009;80:260-266.

103. Warren KR, Postolache TT, Groer ME, Pinjari O, Kelly DL, Reynolds MA. Role of chronic stress and depression in periodontal diseases. *Periodontology 2000* 2014;64:127-138.

104. Solis AC, Lotufo RF, Pannuti CM, Brunheiro EC, Marques AH, Lotufo-Neto F. Association of periodontal disease to anxiety and depression symptoms, and psychosocial stress factors. *Journal of clinical periodontology* 2004;31:633-638.

105. Cordon-Cardo C, Pujadas E, Wajnberg A, et al. COVID-19: Staging of a New Disease. *Cancer Cell* 2020;38:594-597.

106. Carrouel F, Gonçalves LS, Conte MP, et al. Antiviral Activity of Reagents in Mouth Rinses against SARS-CoV-2. *Journal of dental research* 2021;100:124-132.

107. Marouf N, Cai W, Said KN, et al. Association between periodontitis and severity of COVID-19 infection: A case-control study. *Journal of clinical periodontology* 2021.

108. Cirillo N. Reported orofacial adverse effects of COVID-19 vaccines: The knowns and the unknowns. *J Oral Pathol Med* 2021.

109. Ketas TJ, Chaturbhuj D, Cruz-Portillo VM, et al. Antibody responses to SARS-CoV-2 mRNA vaccines are detectable in saliva. *bioRxiv* 2021.

110. Hocková B, Riad A, Valky J, et al. Oral Complications of ICU Patients with COVID-19: Case-Series and Review of Two Hundred Ten Cases. *J Clin Med* 2021;10.

111. Nuno-Gonzalez A, Martin-Carrillo P, Magaletsky K, et al. Prevalence of mucocutaneous manifestations in 666 patients with COVID-19 in a field hospital in Spain: oral and palmoplantar findings. *The British journal of dermatology* 2021;184:184-185.

112. Gherlone EF, Polizzi E, Tetè G, et al. Frequent and Persistent Salivary Gland Ectasia and Oral Disease After COVID-19. *Journal of dental research* 2021:22034521997112.

113. Paradowska-Stolarz AM. Oral manifestations of COVID-19: Brief review. *Dent Med Probl* 2021;58:123-126.
